# Pathways from Family Functioning to Internet Gaming Disorder: The Mediating Role of the Dark Triad

**DOI:** 10.3390/bs14080668

**Published:** 2024-08-02

**Authors:** Danilo Calaresi, Valeria Verrastro, Fiorenza Giordano, Janine Gullo, Valeria Saladino

**Affiliations:** 1 Department of Health Sciences, Magna Graecia University, 88100 Catanzaro, Italy; danilo.calaresi@unicz.it (D.C.); janine.gullo@unicz.it (J.G.); v.saladino@unicz.it (V.S.); 2 Department of Human, Social and Health Sciences, University of Cassino and Southern Lazio, 03043 Cassino, Italy; fiorenza.giordano@unicas.it

**Keywords:** family functioning, Machiavellianism, psychopathy, narcissism, internet gaming disorder, emerging adults

## Abstract

With internet gaming disorder (IGD) becoming more common, there are growing worries about the health of those it affects. This study examines how traits like Machiavellianism, psychopathy, and narcissism might connect family functioning to IGD. The research involved 1190 young adults who answered an online survey, sharing their personal experiences. To examine the mediation effects, latent variable structural equation modeling (SEM) was used, revealing complex relationships among the variables under investigation. Although all direct and indirect paths were statistically significant, the mediation effects of narcissism were positive only when Machiavellianism and psychopathy were not included as parallel mediators, but negative otherwise. The findings suggest that individuals with strong family functioning could be less likely to internalize manipulative behaviors and show a lack of empathy, traits that could contribute to their involvement in IGD. The results underscore the importance of recognizing the multifaceted nature of this phenomenon and provide valuable insights for developing comprehensive strategies to prevent and tackle IGD. Therefore, prevention and intervention efforts should consider the combined influences of family functioning, personality traits, and individual and contextual factors in the online environment to effectively address this problem.

## 1. Introduction

During the digital age, the increase in problematic internet gaming has become a global and pressing issue, attracting the interest of researchers and clinicians around the world. Due to technological progress, online gaming has become more accessible and attractive, leading some individuals to engage in problematic internet gaming behavior [1]. The DSM-5-TR formally acknowledges this conduct as internet gaming disorder (IGD), which involves compulsive and extreme gaming behaviors that result in negative consequences like academic and work problems, social isolation, and mental anguish [2]. The worrying consequences linked to IGD prompt concerns about potential behavioral addiction, with significant implications for public health and overall well-being. Given these worries, it is crucial to explore further the root causes and psychological processes that fuel IGD. Comprehending the reasons and stimuli for excessive gaming can assist in creating precise and successful interventions for tackling this escalating worldwide problem. Recognizing factors that pose risks, like personal characteristics, family interactions, and ways of dealing with stress, can help forecast and stop the development of problematic gaming habits. In addition, identifying the possible connections between IGD and other mental health problems can assist healthcare professionals in offering holistic and cohesive assistance to those facing this type of behavioral dependency.

### 1.1. Family Functioning

The way a family operates is crucial in molding a person’s mental well-being and conduct, significantly impacting their growth and adaptation [3]. Common Italian parenting styles are influenced by cultural values emphasizing family unity and hierarchical structures. Specifically, Italian parents often exhibit an authoritative style, balancing warmth and support with clear expectations and guidance and fostering a nurturing environment while encouraging children to adhere to familial and societal norms [4,5]. Cultural values related to family relationships in Italy underscore the importance of family as a central social unit. Indeed, family loyalty, respect for elders, and a strong sense of duty towards family members are deeply ingrained in Italian culture [4,5]. These values contribute to a family environment that supports emotional strength, interpersonal skills, and adaptive coping strategies, laying the foundation for positive emotional and social development [3].

### 1.2. Family Functioning and Internet Gaming Disorder

According to the literature [6,7], family members who show care, understanding, and effective communication can provide emotional support and connection, potentially decreasing the urge to turn to excessive online gaming for comfort. In environments that encourage growth and development, family members can offer emotional support, empathy, and validation to each other, resulting in better emotional control and less dependence on online distractions. Conversely, dysfunctional family relationships marked by constant conflicts, lack of emotional support, and insufficient supervision can lead to problematic internet behaviors, ultimately affecting an individual’s mental well-being [6,7]. Indeed, during difficult times, people might potentially resort to playing video games on the internet to distract themselves from stressful situations in life or manage emotional issues tied to family conflicts [6,7]. The internet provides a feeling of power, enjoyment, and momentary respite from the stresses faced at home.

### 1.3. The Dark Triad

The dark triad is a combination of three separate characteristics of personality: Machiavellianism, psychopathy, and narcissism [8,9]. Machiavellianism refers to a personality trait characterized by strategic, manipulative, and deceitful behavior aimed at achieving personal goals. Individuals high in Machiavellianism tend to be calculating and pragmatic, often using deception and manipulation to advance their objectives [8,9]. Furthermore, psychopathy is marked by a lack of empathy, guilt, and remorse, combined with impulsive and antisocial behaviors. Individuals with high levels of psychopathy often exhibit a cold and callous demeanor towards others, demonstrating a disregard for social norms and the well-being of others [8,9]. Finally, narcissism involves an inflated sense of self-importance, a strong need for admiration, and a lack of empathy. Individuals with high levels of narcissism have an exaggerated perception of their own achievements and may exploit others to maintain their self-esteem and grandiose self-image [8,9]. 

### 1.4. Family Functioning and the Dark Triad

The familial setting is crucial in influencing a person’s character traits, which may include those related to the dark triad. A supportive and caring family environment, along with effective communication and empathy, can promote positive personality development and serve as a shield against the development of dark triad characteristics [10,11]. In settings that provide support, people are more likely to acquire positive behaviors and skills in managing emotions, which can offset the negative traits associated with the dark triad [11,12]. Having caring and empathetic adults around can promote the growth of compassion, generosity, and accountability towards others. Conversely, maladaptive characteristics linked to the dark triad may be impacted by dysfunctional family dynamics and negative interpersonal interactions [10,13]. Kids raised in homes with abusive or neglectful behaviors might show manipulative and indifferent traits in their relationships with others due to negative interaction patterns [12,13]. Experiencing negative family dynamics during childhood can impact an individual’s perspective on relationships, resulting in a skewed understanding of social interactions. Both family dynamics and the dark triad have a mutual influence on each other, forming a bilateral relationship [11,13]. Unfavorable family settings can worsen current dark personality traits, causing an increase in destructive actions [10,12]. Conversely, having dark triad traits in the family can cause conflicts and disruptions in the family dynamic [14,15]. 

#### Family Functioning and the Individual Role of the Dark Triad Traits

In the context of family functioning, the individual dark triad traits can interact in complex ways. Individuals high in Machiavellianism might strategically influence family dynamics to serve their own interests, often through deception or manipulation [12,13]. This behavior can lead to increased conflict and instability within the family, as these individuals may exploit family members for their gain, causing trust issues and interpersonal strife [12,13]. Those with high levels of psychopathy may disregard the feelings and needs of others which can undermine emotional support within the family, leading to strained relationships and a lack of mutual trust and understanding [11,13]. This disregard for social norms and family welfare can contribute to a toxic family environment, which may exacerbate conflicts and emotional distress [11,13]. The self-centeredness of narcissistic individuals can create significant friction within the family, as narcissistic individuals may neglect or belittle the needs of others in favor of maintaining their own self-esteem [11,14]. The resultant strain on family relationships can lead to persistent conflicts and a lack of harmony, further exacerbating issues related to family functioning [11,14]. 

### 1.5. The Dark Triad and Internet Gaming Disorder

Researchers are currently investigating the possible correlation between the dark triad traits and problematic internet behaviors to determine if individuals with these traits are more likely to partake in excessive and detrimental online activities. Initial results indicate that the dark triad characteristics could be linked to a higher chance of problematic internet usage. Specifically, the relationship between the dark triad traits and IGD can be understood through the lens of impulsivity, emotional regulation, social manipulation, and the need for validation [14,16,17]. Each of these traits influences gaming behavior in distinct but overlapping ways, potentially leading to excessive gaming as a form of escape, gratification, or social engagement [14,15,18].

#### Internet Gaming Disorder and the Individual Role of the Dark Triad Traits

More precisely, people with high scores in those characteristics might use the internet for asserting dominance, control, and self-improvement [16,18]. It is reasonable to consider that individuals with high levels of Machiavellianism might use dishonest strategies, such as cheating or taking advantage of less powerful players, in order to outperform others in online gaming [14,15]. This kind of conduct can result in a negative gaming experience for both the manipulator and the people they are influencing. Moreover, people with psychopathic characteristics could exhibit a deficiency in empathy and guilt, leading to unfriendly and hostile behavior in online gaming societies. They might partake in trolling, cyberbullying, or other disruptive and harmful behaviors without feeling remorse [14,17]. Those displaying narcissistic tendencies may become overly fixated on their accomplishments within the game and continually seek praise from others for their gaming abilities. This desire for acknowledgment and approval might lead them to prioritize online interactions over their actual duties and connections in reality [15,17].

### 1.6. Aims of the Study

IGD is increasingly worrying on a worldwide scale, with the possibility of harming individual mental health, social connections, and general happiness. It is important to understand the reasons behind problematic gaming behavior in order to create successful prevention and intervention strategies to tackle this problem. The way a family operates is highly influential in determining how individuals behave and develop psychologically. Studying the correlation between family dynamics and IGD can offer an understanding on how family settings might influence or lessen the likelihood of excessive gaming. This information can assist families in recognizing possible risk factors and enhancing their support systems for their loved ones. The dark triad traits involve manipulative tendencies, lack of empathy, and a craving for power and dominance. Comprehending how these characteristics play a role in the connection between family functioning and IGD can provide insights into the fundamental reasons why individuals with these traits resort to excessive gaming as a way to cope or escape. Investigating how the dark triad traits influence problematic gaming behavior can enhance psychology’s knowledge of the intricate relationship between personality traits and online activities. This study can enhance the increasing knowledge base about the psychological dimensions of gaming and personality, providing valuable insights to scholarly works and deepening our comprehension of human interactions in digital spaces.

**Figure 1:**
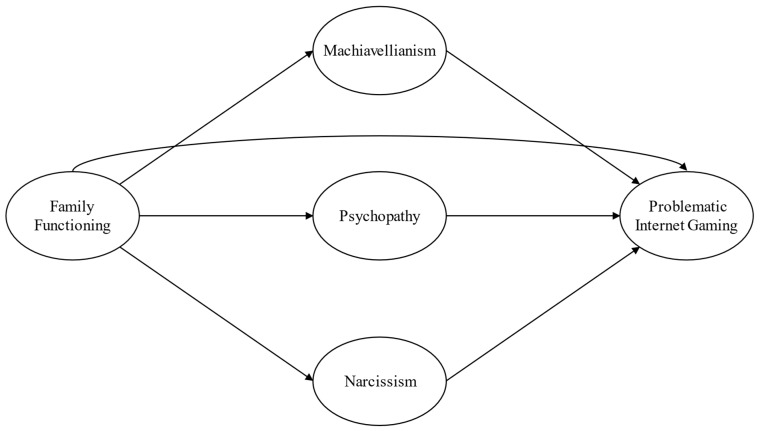
Hypothesized main model.

Expanding on the points mentioned earlier, the main aim of this study is to explore the potential mediating roles of Machiavellianism, psychopathy, and narcissism in the associations between family functioning and IGD (Figure 1). We hypothesize the following:

**H1.**  *Poor family functioning will be positively associated with higher levels of IGD.*

**H2.**  *Machiavellianism, psychopathy, and narcissism will mediate the relationship between family functioning and IGD.*

## 2. Materials and Methods

### 2.1. Participants

The study included a convenience sample of 1190 young adults in Italy, evenly split between 595 women and 595 men. The age of the participants ranged from 18 to 25 years (M = 21.76, SD = 2.18). The participants were recruited through online channels, utilizing several social networks. The criteria for inclusion encompassed individuals aged between 18 and 25 years, possessing fluency in Italian, having engaged in video gaming within the last six months, and maintaining an average of 7 h of video gaming per week. Regarding educational attainment, 11% of participants had completed middle school, 56% held a high school diploma, 25% had obtained a university degree, and 8% had pursued postgraduate studies. With respect to occupational status, 35% of participants were students, 35% were unemployed, 23% were employed, and 7% were self-employed. In terms of marital status, 42% of participants were single, 41% were engaged, 9% were cohabiting, and 8% were married.

### 2.2. Procedures

The current study adhered to ethical guidelines outlined in the Declaration of Helsinki and the Italian Association of Psychology (AIP). Approval for the study was granted by the Institutional Review Board of the Institute for the Study of Psychotherapy, School of Specialization in Brief Psychotherapies with a Strategic Approach (reference number: ISP-IRB-2023-5). Participants were invited to take part in a comprehensive online survey via Google Forms. Their completion of the survey was required to ensure the collection of comprehensive data; hence, there were no missing data (e.g., [19]). Only individuals who provided informed consent were included in the study, and their participation was voluntary, without any form of compensation. The privacy and confidentiality of the participants were strictly upheld throughout all stages of the research process.

### 2.3. Measures

#### 2.3.1. Family Functioning

Family functioning was assessed using the Italian version of the General Functioning Subscale of the McMaster Family Assessment Device (FAD), developed by Epstein et al. [20] and adapted by Roncone et al. [21]. The General Functioning Subscale refers to the overall health/pathology of the family and is composed of 12 items, such as “Making decisions is a problem for our family”. Each item was measured on a 4-point Likert scale, ranging from 1 (strongly agree) to 4 (strongly disagree). To calculate the overall level of family functioning, the scores for the 12 items were averaged, with higher scores indicating higher levels of general functioning. In this study, the internal consistency of the scale was found to be good, as indicated in Table 1.

#### 2.3.2. Dark Triad

The Dark Triad Dirty Dozen (DTDD) scale, developed by Jonason and Webster [22] and validated in Italian by Schimmenti et al. [8], was employed to assess dark personality traits. This measurement scale comprises 12 items, with each of the three traits within the dark triad (narcissism, psychopathy, and Machiavellianism) being measured by four items. For example, items measuring narcissism include statements such as “I tend to want others to admire me”. Items assessing psychopathy include statements like “I tend to lack remorse”, while Machiavellianism is measured using items such as “I tend to manipulate others to get my way”. Higher scores on each subscale indicate a greater presence of the corresponding personality trait. In this study, the internal consistency of the DTDD scale was found to be good, as shown in Table 1.

#### 2.3.3. Internet Gaming Disorder

To measure IGD, the study utilized the Italian version of the Internet Gaming Disorder Scale—Short-Form (IGDS9-SF) developed by Pontes and Griffiths [23] and adapted by Monacis et al. [24]. The IGDS9-SF is a 9-item self-report scale which assesses the severity of gaming-related issues and identifies individuals who may require further assessment or intervention. An example item is “Do you systematically fail when trying to control or cease your gaming activity?”. Participants were asked to rate each item on a 5-point Likert scale, ranging from 1 (never) to 5 (very often). Higher scores indicate higher levels of IGD. In this study, the internal consistency of the IGDS9-SF demonstrated good reliability, as shown in Table 1.

### 2.4. Statistical Analyses

Descriptive statistics and correlation analyses were conducted using the IBM SPSS 27 software. The primary analyses utilized the lavaan package in RStudio 2023.09.1 +494 to perform the subsequent statistical procedures.

To assess the potential impact of gender, we used a multivariate analysis of variance (MANOVA). This analysis examined the effects of gender on multiple dependent variables, including family functioning, Machiavellianism, psychopathy, narcissism, and IGD. When significant multivariate effects of gender were found, we performed additional univariate analyses and pairwise comparisons, applying Bonferroni correction to control for multiple comparisons.

To examine the mediation models, a latent variable structural equation modeling (SEM) technique was utilized. The first model included family functioning as the predictor variable, Machiavellianism, psychopathy, and narcissism as the mediator variables, and IGD as the outcome variable. The second model included family functioning as the predictor variable, Machiavellianism as the only mediator variable, and IGD as the outcome variable. The third model included family functioning as the predictor variable, psychopathy as the only mediator variable, and IGD as the outcome variable. The fourth model included family functioning as the predictor variable, narcissism as the only mediator variable, and IGD as the outcome variable. In assessing the significance of the indirect effects within the mediation models, the bias-corrected confidence interval method was utilized. This was generated through bootstrap resampling with 5000 resamples, allowing for the estimation of confidence intervals and determining statistical significance.

## 3. Results

### 3.1. Descriptive Statistics and Correlations

Table 1 presents the descriptive statistics and correlations among the variables examined in this study. The obtained means in this investigation align with previous research findings [8,25,26].

Initial analyses were carried out to examine the potential influence of gender on the study variables. A MANOVA was conducted, which showed significant multivariate effects of gender, indicated by Wilks’ λ = 0.97 (F(5, 1184) = 7.91, *p* < 0.001, ηp^2^ = 0.03). Subsequent univariate analyses indicated that gender had effects on Machiavellianism (F(1, 1188) = 18.68, *p* < 0.001, ηp^2^ = 0.02), psychopathy (F(1, 1188) = 25.63, *p* < 0.001, ηp^2^ = 0.02), narcissism (F(1, 1188) = 6.03, *p* = 0.01, ηp^2^ = 0.01), and IGD (F(1, 1188) = 29.98, *p* < 0.001, ηp^2^ = 0.03). Specifically, men exhibited higher levels of Machiavellianism, psychopathy, narcissism, and IGD compared to women. Given the significant multivariate effects, gender was included as a control variable in the main analyses.

### 3.2. Mediation Models

The proposed models, which employed structural equation modeling (SEM) with latent variables, were assessed for their goodness of fit using the collected data.

**Figure 2:**
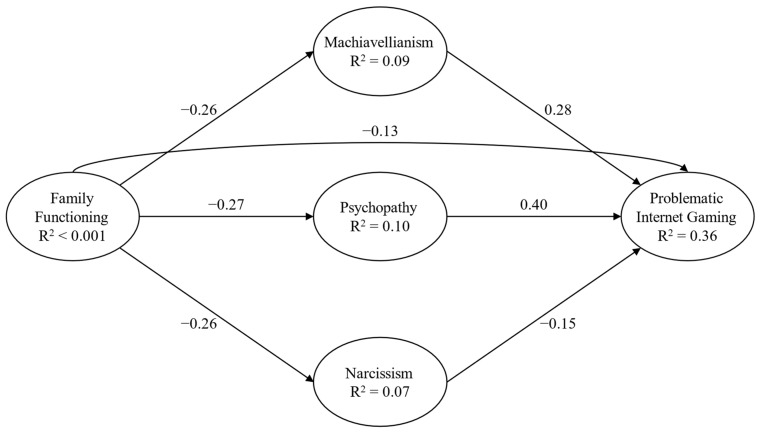
Structural Model 1. Note: only direct paths are reported for presentation and clarity purposes; paths from background variables were not presented for presentation and clarity purposes; and parcels were not presented for presentation and clarity purposes.

The first model (Figure 2) showed a good fit: χ^2^(90) = 346.94, *p* < 0.001, CFI = 0.98, RMSEA = 0.05 (90% CI = 0.04–0.05), and SRMR = 0.04. Significant relationships, both direct and indirect, were observed between all the study variables (Table 2).

**Figure 3:**
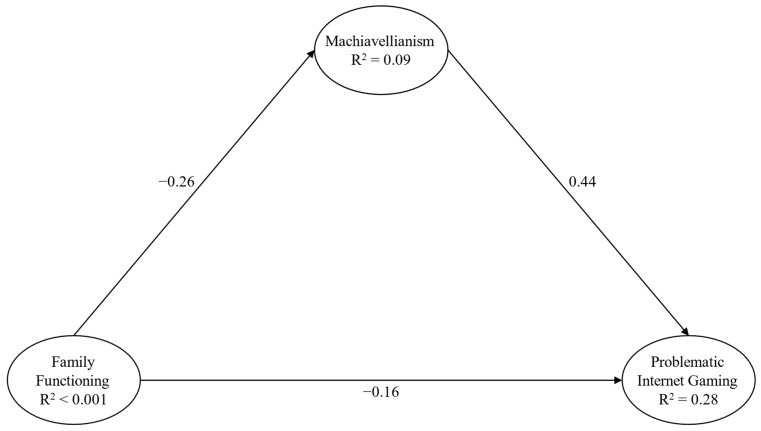
Structural Model 2 Note: only direct paths are reported for presentation and clarity purposes; paths from background variables were not presented for presentation and clarity purposes; and parcels were not presented for presentation and clarity purposes.

The second model (Figure 3) exhibited a satisfactory fit: χ^2^(30) = 104.16, *p* < 0.001, CFI = 0.99, RMSEA = 0.05 (90% CI = 0.04–0.06), and SRMR = 0.03. Significant paths, both direct and indirect, were highlighted between all the study variables (Table 3).

**Figure 4:**
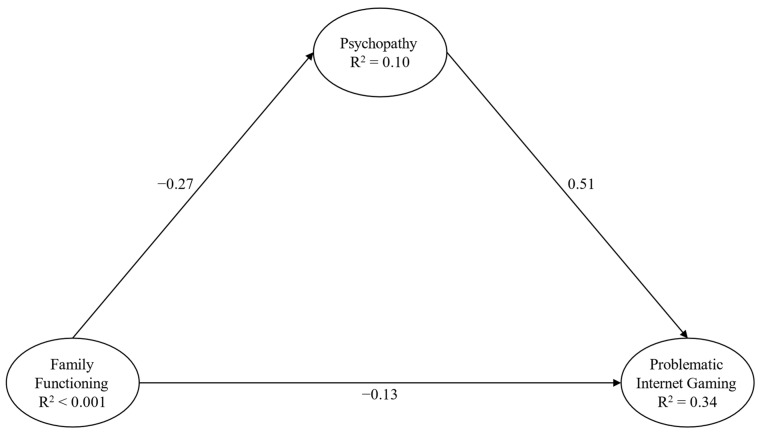
Structural Model 3 Note: only direct paths are reported for presentation and clarity purposes; paths from background variables were not presented for presentation and clarity purposes; and parcels were not presented for presentation and clarity purposes.

The third model (Figure 4) highlighted a good fit: χ^2^(30) = 48.50, *p* = 0.02, CFI = 0.997, RMSEA = 0.02 (90% CI = 0.01–0.03), and SRMR = 0.02. Significant paths, both direct and indirect, were highlighted between almost all the study variables (Table 4).

**Figure 5:**
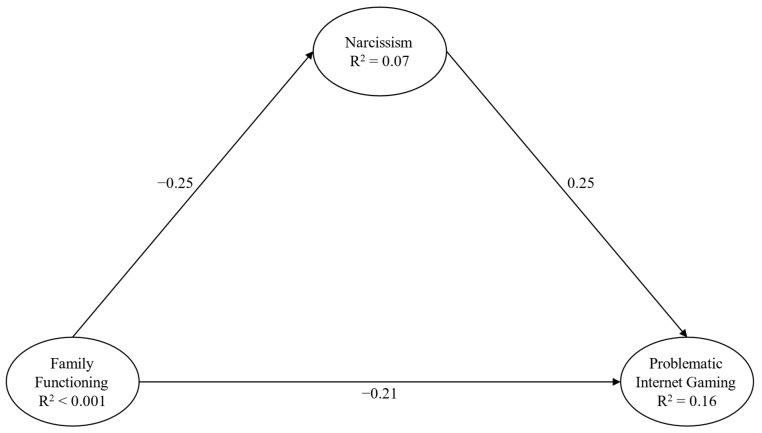
Structural Model 4 Note: only direct paths are reported for presentation and clarity purposes; paths from background variables were not presented for presentation and clarity purposes; and parcels were not presented for presentation and clarity purposes.

The fourth model (Figure 5) underlined a satisfactory fit: χ^2^(30) = 139.70, *p* < 0.001, CFI = 0.98, RMSEA = 0.06 (90% CI = 0.05–0.07), and SRMR = 0.04. Significant paths, both direct and indirect, were highlighted between almost all the study variables (Table 5).

## 4. Discussion

The main aim of this research was to investigate how Machiavellianism, psychopathy, and narcissism may act as mediators between family functioning and IGD. The study’s results offer a valuable understanding of the complex relationships between family functioning, the dark triad traits, and behaviors associated with internet gaming.

More precisely, we found that higher levels of family functioning were linked to lower likelihood of engaging in IGD among emerging adults. This is consistent with previous research that emphasizes the adverse effects of dysfunctional family environments on the social and psychological growth of children [3]. Additionally, our research discovered a significant mediating impact of the dark triad personality traits on the correlation between family functioning and IGD. This indicates that the dark triad characteristics serve as pathways by which family dynamics impact IGD in young adults. Our results support past studies that have individually explored the connections between the dark triad traits and IGD [14,15], and the relationship between family functioning and IGD [10,11]. Nevertheless, our research adds to the field by offering empirical proof for the involvement of the dark triad traits in this connection. Furthermore, our research also revealed intricate connections among the factors being studied, suggesting that the interaction between family dynamics, the dark triad characteristics, and IGD is complex and warrants additional investigation.

Individuals who grow up in dysfunctional family environments face a myriad of challenges that can profoundly impact their well-being and emotional development. The elevated stress, frequent conflicts, and emotional struggles in such environments create a complex emotional landscape for these individuals to navigate [3]. As a result, some individuals may develop coping mechanisms to adapt to these difficult family dynamics. One such coping mechanism is observed in individuals with Machiavellian traits, who are known for their strategic and manipulative tendencies. The skillset honed through Machiavellianism may offer them a way to navigate the complexities of their family life [12,13]. IGD then becomes a refuge for these individuals, a place where they can exercise control and seek escape from the challenges they experience in their real-life environment. Within the virtual world of online gaming, where social interactions, alliances, and competitions abound, Machiavellian individuals find fertile ground to exploit others and gain advantages [10,12]. This can become especially relevant when their dysfunctional family dynamics fail to provide positive role models or healthy social connections, making online gaming an appealing platform to assert control and satisfy their strategic inclinations.

Similarly, psychopathic individuals, with their callous and unemotional traits, may find solace in IGD as a coping mechanism to cope with the emotional difficulties they encounter in their family life [11,13]. The virtual realm of gaming offers a sanctuary to numb or escape from these challenging emotional experiences, allowing them to avoid confronting the negative feelings associated with their upbringing. Psychopathy is also linked to a craving for excitement and stimulation, making fast-paced and action-packed games particularly enticing for these individuals [14,15]. Within dysfunctional family environments that lack sufficient stimulation or excitement, the allure of excessive gaming becomes even more attractive. In addition, their natural inclination for power and control is well-suited for online gaming, where they can demonstrate dominance and wield authority over rivals [14,17]. The online arena transforms into a platform where individuals can satisfy their desire for dominance and authority, meeting a longing that might not be fulfilled in their actual familial environment.

For individuals with narcissistic traits, IGD offers a canvas for their self-centered inclinations and a means to compensate for perceived inadequacies or negative emotions stemming from their family life [10,15]. The virtual world of gaming becomes a stage where they receive the adoration and recognition they crave, temporarily boosting their fragile self-esteem. Dysfunctional family dynamics that fail to provide sufficient positive reinforcement or validation can fuel the need for external affirmation, leading these individuals to seek validation through excessive gaming [11,14]. The desire for self-enhancement and attention-seeking behaviors draws them to multiplayer or competitive settings, where they can showcase their skills and accomplishments to a larger audience, garnering the admiration they seek. The virtual stage offers them a platform to express their grandiose fantasies and aspirations, creating avatars that embody their idealized selves [15,17]. Within the immersive communities of online gaming, opportunities arise for them to exploit or manipulate others for their benefit, further reinforcing their sense of self-importance. The dynamic and competitive nature of online gaming aligns with their yearning to be perceived as superior, especially if they feel unacknowledged or undervalued within their family environment [13,14]. The detachment from real-life consequences in the virtual world enables them to express their self-centered behaviors without the burden of empathy or social expectations.

The discrepancy between the positive correlation found in the Pearson’s correlation analysis between narcissism and IGD and the negative relationship observed in the mediation model can be attributed to various theoretical factors. One possible explanation is the influence of a third variable (Machiavellianism and psychopathy in this case), which can alter the relationship between the two variables. The strong positive relationships between Machiavellianism, psychopathy, and cyberbullying behaviors may overshadow the relationship between narcissism and cyberbullying behaviors when considered together in the mediation model. This could lead to a negative relationship between narcissism and IGD when mediated by narcissism. Moreover, each dark triad trait may have distinct mediation effects on the relationship between family functioning and IGD. While the fourth model indicates a positive mediation effect for narcissism when examined independently, the more complex model including all three traits shows narcissism acting as a negative mediator, inhibiting the direct relationship between family functioning and IGD. These findings underscore the significance of considering the combined effects of dark triad traits when studying their mediation effects on the link between family functioning and IGD. The study’s results align with recent research indicating that while narcissism seems to have a positive relationship with problematic internet behaviors like cyberbullying, its mediating effects can become insignificant or negative when accounting for the other two dark triad traits [27,28]. However, it is crucial to interpret the finding regarding the negative mediating effect of narcissism cautiously, as the effect size was small, similar to the latter study [28]. Furthermore, the literature on narcissism’s mediating effects reveals intricate relationships, with different outcomes observed in various dimensions of narcissism. For example, covert narcissism and overt narcissism or pathological narcissism vulnerability and grandiosity can lead to different results [29,30]. While the present study contributes to understanding these complexities, further research is needed to gain deeper insights into these phenomena.

Finally, the significant gender differences identified in our MANCOVA highlight the necessity of incorporating gender as a critical factor when examining the relationships between family dynamics, dark triad traits, and IGD. Men’s higher levels of Machiavellianism, psychopathy, and narcissism suggest that they may be more prone to using manipulative or exploitative strategies and seeking validation through gaming [31,32]. This may imply that interventions for IGD could benefit from being tailored to address these specific traits more effectively in men.

## 5. Conclusions

### 5.1. Limitations

Our research has several limitations that need to be addressed. The use of a cross-sectional design restricts our ability to determine causality between variables. Longitudinal studies tracking participants over time would provide a better understanding of these relationships and offer stronger evidence for the observed correlations. Furthermore, relying on self-reported data introduces potential bias, as participants’ perceptions and responses may affect the accuracy of the information. Future research should integrate multiple data sources, including objective measures and external feedback, to enhance the reliability and comprehensiveness of the findings. Finally, our study used only online data collection methods, which may exclude individuals without internet access or those less likely to participate in online surveys. To obtain a more representative sample, future studies should consider alternative data collection methods, such as face-to-face interviews or non-online sources, to ensure broader inclusivity and a more accurate reflection of the target population.

### 5.2. Clinical Implications and Future Research

The findings of this research offer an important understanding into the complex relationships among family dynamics, the dark triad traits, and IGD. The mediation techniques used in this study reveal the underlying factors that lead to the development of IGD in young adults.

Clinicians are advised to consider the presence of these traits when addressing issues related to family dynamics and gaming behaviors. Recognizing how Machiavellianism, psychopathy, and narcissism act as mediators can lead to more tailored interventions. For those with Machiavellian or psychopathic traits, interventions might target manipulative behaviors both in gaming and real life. In contrast, individuals with narcissistic traits may benefit from strategies that help them build self-worth and find validation through healthier channels rather than excessive gaming. The findings also underscore the potential benefits of family therapy. Since family functioning plays a crucial role in IGD, therapeutic approaches that address dysfunctional family dynamics and teach better stress management and conflict resolution could be particularly effective. 

Looking ahead, incorporating diverse age groups in research could shed light on how the influence of dark triad traits evolves over different life stages, helping to pinpoint key moments for intervention and prevention. Additionally, examining these relationships across various cultural contexts might reveal whether the connections are universally applicable or specific to certain cultures. Exploring the mechanisms through which dark triad traits mediate the relationship between family dynamics and IGD could further refine intervention strategies. Ultimately, understanding these complexities will aid in developing comprehensive treatment plans that address the multifaceted nature of gaming disorders.

## Figures and Tables

**Table 1:** Descriptive analyses and correlations.

	M	SD	α	ω	1	2	3	4
1. Family Functioning	2.88	0.71	0.89	0.89	-	-	-	-
2. Machiavellianism	0.83	0.96	0.86	0.86	−0.23 *	-	-	-
3. Psychopathy	0.92	0.88	0.80	0.80	−0.23 *	0.59 *	-	-
4. Narcissism	1.47	1.08	0.83	0.84	−0.22 *	0.61 *	0.44 *	-
5. Problematic Internet Gaming	1.46	0.81	0.94	0.94	−0.26 *	0.44 *	0.49 *	0.27 *

Note: * *p* < 0.01.

**Table 2:** Path estimates, SEs, and 95% CIs of Structural Model 1.

	β	*p*	SE	CI	CI
				LL	UL
Direct Effect					
Family Functioning → Machiavellianism	−0.26	<0.001	0.04	−0.44	−0.27
Family Functioning → Psychopathy	−0.27	<0.001	0.04	−0.42	−0.26
Family Functioning → Narcissism	−0.26	<0.001	0.05	−0.46	−0.26
Family Functioning → Problematic Internet Gaming	−0.13	<0.001	0.03	−0.22	−0.13
Machiavellianism → Problematic Internet Gaming	0.28	<0.001	0.07	0.13	0.40
Psychopathy → Problematic Internet Gaming	0.40	<0.001	0.06	0.29	0.50
Narcissism → Problematic Internet Gaming	−0.15	0.01	0.05	−0.22	−0.04
Indirect Effect via Machiavellianism					
Family Functioning → Problematic Internet Gaming	−0.04	0.003	0.01	−0.07	−0.02
Indirect Effect via Psychopathy					
Family Functioning → Problematic Internet Gaming	−0.05	<0.001	0.02	−0.09	−0.03
Indirect Effect via Narcissism					
Family Functioning → Problematic Internet Gaming	0.02	0.03	0.01	0.01	0.04
Total Effect	−0.20	<0.001	0.05	−0.34	−0.14

Note: β standardized regression coefficients; *p* level of significance; SE standard error; CI confidence interval; LL lower limit; and UL upper limit.

**Table 3:** Path Estimates, SEs and 95% CIs of Structural Model 2.

	β	*p*	SE	CI	CI
				LL	UL
Direct Effect					
Family Functioning → Machiavellianism	−0.26	<0.001	0.14	−0.44	−0.27
Family Functioning → Problematic Internet Gaming	−0.16	<0.001	0.03	−0.26	−0.13
Machiavellianism → Problematic Internet Gaming	0.44	<0.001	0.04	0.32	0.49
Indirect Effect via Machiavellianism					
Family Functioning → Problematic Internet Gaming	−0.07	<0.001	0.01	−0.10	−0.05

Note: β standardized regression coefficients; *p* level of significance; SE standard error; CI confidence interval; LL lower limit; and UL upper limit.

**Table 4:** Path Estimates, SEs and 95% CIs of Structural Model 3.

	β	*p*	SE	CI	CI
				LL	UL
Direct Effect					
Family Functioning → Psychopathy	−0.27	<0.001	0.04	−0.42	−0.25
Family Functioning → Problematic Internet Gaming	−0.13	<0.001	0.03	0.23	−0.10
Psychopathy → Problematic Internet Gaming	0.51	<0.001	0.04	0.43	0.60
Indirect Effect via Psychopathy					
Family Functioning → Problematic Internet Gaming	−0.07	<0.001	0.02	−0.12	−0.05

Note: β standardized regression coefficients; *p* level of significance; SE standard error; CI confidence interval; LL lower limit; and UL upper limit.

**Table 5:** Path Estimates, SEs and 95% CIs of Structural Model 4.

	β	*p*	SE	CI	CI
				LL	UL
Direct Effect					
Family Functioning → Narcissism	−0.25	<0.001	0.05	−0.48	−0.27
Family Functioning → Problematic Internet Gaming	−0.21	<0.001	0.04	−0.32	−0.19
Narcissism → Problematic Internet Gaming	0.25	<0.001	0.03	0.15	0.28
Indirect Effect via Narcissism					
Family Functioning → Problematic Internet Gaming	−0.05	<0.001	0.01	−0.07	−0.04

Note: β standardized regression coefficients; *p* level of significance; SE standard error; CI confidence interval; LL lower limit; and UL upper limit.

## Data Availability

The data presented in this study are available on request from the corresponding author (the data are not publicly available due to privacy and ethical restrictions).
